# Flexible Topical Hydrogel Patch Loaded with Antimicrobial Drug for Accelerated Wound Healing

**DOI:** 10.3390/gels9070567

**Published:** 2023-07-12

**Authors:** Sana Saeed, Kashif Barkat, Muhammad Umer Ashraf, Maryam Shabbir, Irfan Anjum, Syed Faisal Badshah, Muhammad Aamir, Nadia Shamshad Malik, Akash Tariq, Riaz Ullah

**Affiliations:** 1 Faculty of Pharmacy, The University of Lahore, Lahore 54000, Pakistan; sanasaeed6@hotmail.com (S.S.); muhammad.ashraf3@pharm.uol.edu.pk (M.U.A.); maryam.shabbir@pharm.uol.edu.pk (M.S.); faisalpharm8@gmail.com (S.F.B.); muhammad.aamir@pharm.uol.edu.pk (M.A.); 2 Department of Basic Medical Sciences, Shifa College of Pharmaceutical Sciences, Shifa Tameer-e-Millat University, Islamabad 44000, Pakistan; anjuum95@yahoo.com; 3 Faculty of Pharmacy, Capital University of Science and Technology (CUST), Islamabad 44000, Pakistan; nadiashamshad@gmail.com; 4 Xinjiang Institute of Ecology and Geography, Chines Academy of Sciences, Urumqi 830011, China; akash.malik786@mails.ucas.ac.cn; 5 Department of Pharmacognosy, College of Pharmacy, King Saud University, Riyadh 11451, Saudi Arabia; rullah@ksu.edu.sa

**Keywords:** sodium alginate, hydroxyethylcellulose, hydrogel patch, neomycin, skin deposition, wound healing

## Abstract

A hydrogel topical patch of neomycin was developed by using sodium alginate (SA) and hydroxyethylcellulose (HEC) as polymers. Free radical polymerization in an aqueous medium was initiated by using acrylic acid (AA) and N,N′-methylenebisacrylamide (MBA). Prepared hydrogels were characterized for pH sensitivity and sol–gel analysis. In addition, the effect of reactant contents on the developed formulation was evaluated by swelling behavior. SEM assay showed the rough structure of the hydrogel-based polymeric matrix, which directly enhances the ability to uptake fluid. FTIR spectra revealed the formation of a new polymeric network between reactant contents. TGA and DSC verified that fabricated polymeric patches were more thermodynamically stable than pure components. Gel fractions increased with increases in polymer, monomer, and cross-linker contents. The swelling study showed the pH-dependent swelling behavior of patches at pH 5.5, 6.5, and 7.4. The release pattern of the drug followed zero-order kinetics, with diffusion-controlled drug release patterns according to the Korsmeyer–Peppas (KP) model. Ex vivo studies across excised rabbit skin verified the drug retention in the skin layers. The hydrogel patch effectively healed the wounds produced on the rabbit skin, whereas the formulation showed no sign of irritation on intact skin. Therefore, neomycin hydrogel patches can be a potential candidate for controlled delivery for efficient wound healing.

## 1. Introduction

The skin is the largest organ in the human body, comprising approximately 90% of body weight and volume. It provides protection against environmental stimuli such as moisture, heat, cold, and pressure, and serves as the primary physical barrier against invading pathogens and diseases [1,2]. A wound is a disruption of the typical anatomy and physiology of the skin. It is brought on by a variety of external events, including burns, infections, surgeries, and thermal or physical injuries [3]. According to how long it takes for a wound to heal, it can be classified as acute or chronic, whilst burns are also categorized according to how badly the skin layer has been burned. Homeostasis, inflammation, proliferation, and remodeling are the four fundamental stages of wound healing. Cellular signaling from the microenvironment controls this complex biological process. In all phases of wound healing, immune cells like neutrophils, macrophages, and leukocytes play a crucial role [4].

Due to their excellent moisture-retention properties, hydrogel-based dressings are an excellent option for wound healing and cause less distress when removed [5]. Hydrogels are 3-D networks of hydrophilic polymers that can contain a significant amount (90%) of water and biological fluids without dissolving. By reducing the first pass effect and dosage frequency in contrast to conventional drug delivery systems, hydrogels have contributed significantly to the development of controlled-release topical drug delivery systems. Because hydrogels are sensitive to external stimuli such as temperature, ionic strength, and pH, they are referred to as smart or intelligent systems. They are generated when hydrophilic polymers are physically or chemically cross-linked [6]. Hydrogel biomaterials with inherent antimicrobial properties offer a desirable and useful solution to wound healing [7].

Recently, Cheng et al. (2022) reported the formation of a dendritic hydrogel patch that showed marked antimicrobial activity against various pathogens using synthetic compounds. However, natural polymers have been widely used in the development of hydrogel dressings and are an excellent alternative to synthetic polymers due to optimal features such as biodegradability, flexibility, and economic availability [8]. Numerous researchers have adopted the use of polymers to exert antimicrobial activity as a well-established phenomenon. Sodium alginate (SA) is a polymer derived naturally from brown algae. It plays an essential role in the healing of burns and wounds as a result of its biocompatibility and ability to mend tissues without causing adverse effects [9]. Similarly, due to its distinctive properties, including biocompatibility, emulsifying, swelling, and film-forming capacity. Not only has it been used in controlled-release applications of drugs or pesticides, but also as a thickening agent, gelling agent, and colloidal stabilizer in the biotechnology industry. When in contact with calcium ions or glutaraldehyde, alginate salts are known to form a reticulated structure, which has been used to create sustained-release particulate systems for a variety of drugs, portions, and even cells [10]. Hydroxyethylcellulose (HEC) is one of the most significant polymers utilized in the fabrication of hydrogel patches. Due to its excellent mechanical strength and great capacity to hold water, HEC has emerged to have a wide range of uses in the therapy of wounds [11]. It can also be used as a thickener and has good resistance to dry heat [12]. Acrylic acid is composed of carboxylic groups, which upon ionization at higher pH than its pKa value, results in high electrostatic repulsion. This electrostatic repulsion supports significant swelling of the system, which alternatively enhances the significant release of the drug from the system.

Neomycin is classified as BCS class III. It is soluble in both hot and cold water and has high solubility and low permeability. After oral administration, the bioavailability of neomycin is 2%. It is administered orally to patients with hepatic coma and renal encephalopathy despite its limited absorption. Neomycin is used topically for burns and wound healing because it is a nephrotoxic drug that causes systemic toxicity when taken orally [13]. Renal excretion accounts for 1% of the total, and plasma half-life is 2 h. Neomycin has a molecular weight of 441.6 g/mol, volume of distribution is 0.25 L/kg, and plasma protein binding is 20%. For therapeutic purposes, 1 g of medication is taken orally every 4 h, and 100 mg is used topically 2–3 times each day. Such properties of neomycin make it a prime option to serve as a model drug for the creation of hydrogel topical patches for sepsis and wound healing [14].

The current study aimed to create a neomycin topical hydrogel patch using polymers having wound healing properties such as SA and HEC. The physicochemical parameters and sol–gel analysis of the created patch were studied. The primary purpose of the study was to determine the sustained-release properties of the patch over 72 h and drug retention in the skin. The patches were applied to a wounded rabbit after establishing hydrogel system compatibility by FTIR, XRD, and DSC, and the degree of healing was compared to the marketed formulation. 

## 2. Results and Discussion

### 2.1. Macro-Morphological and Micro-Morphological Evaluation of Topical Hydrogel Patch

**Figure 1:**
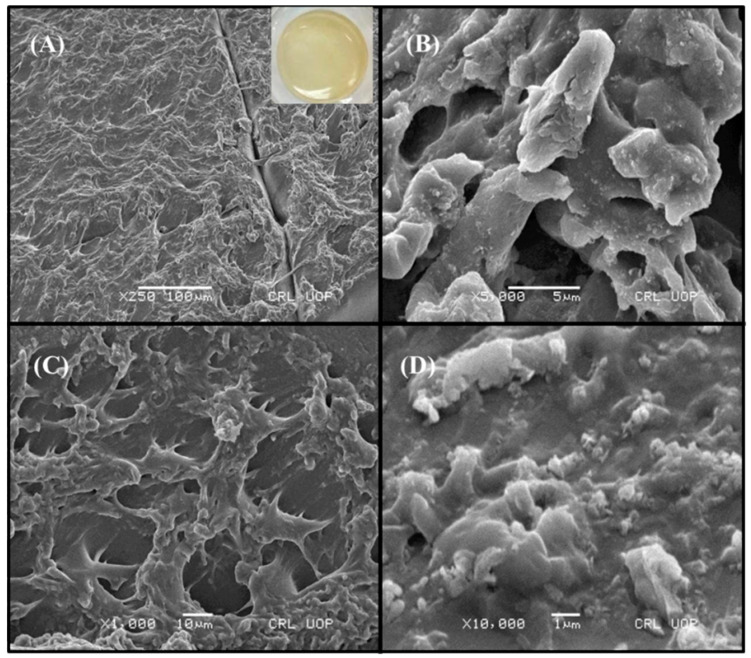
Macro-morphological structure (embedded image), SEM analysis of (**A**,**B**) surface, and (**C**,**D**) cross-sectional view of topical hydrogel patch (HECA-12).

The patches prepared with varying concentrations of polymers (HEC and SA), monomers (AA), and cross-linkers (MBA) showed a brownish tinge upon drying, as shown in Figure 1A (embedded image). As per SEM analysis, the micrographs of the patch showed a rough and coarse surface morphology. Such properties result in the formation of a surface, which is helpful for increased water entrance within the polymeric network. At higher magnification (Figure 1B), a higher number of open channels and pores were observed that supported the three-dimensional matrix. This porosity facilitates the permeation of water into the polymeric network. Cross-sectional images of the neomycin-loaded topical patch (Figure 1C,D) revealed a highly porous and coarse structure. This porous nature of the formulated hydrogel network provides a high number of pores for the placement of drug molecules. When a porous structure swells, it opens up huge pores that might facilitate the loading of drug molecules into the formed pockets. The outcomes obtained were quite promising in terms of drug entrapment in the formulation, thus serving as an effective wound treatment when applied to wound and infection sites [15].

### 2.2. Physical Characteristics of Topical Hydrogel Patches

The polymeric cross-linked topical hydrogel patches exhibited uniform values for weight, thickness, and folding endurance, as given in Table 1. A lower value of SD demonstrated that the process was reproducible with minimum deviations from the desired values [16,17]. 

### 2.3. FTIR Analysis of Topical Hydrogel Patch

**Figure 2:**
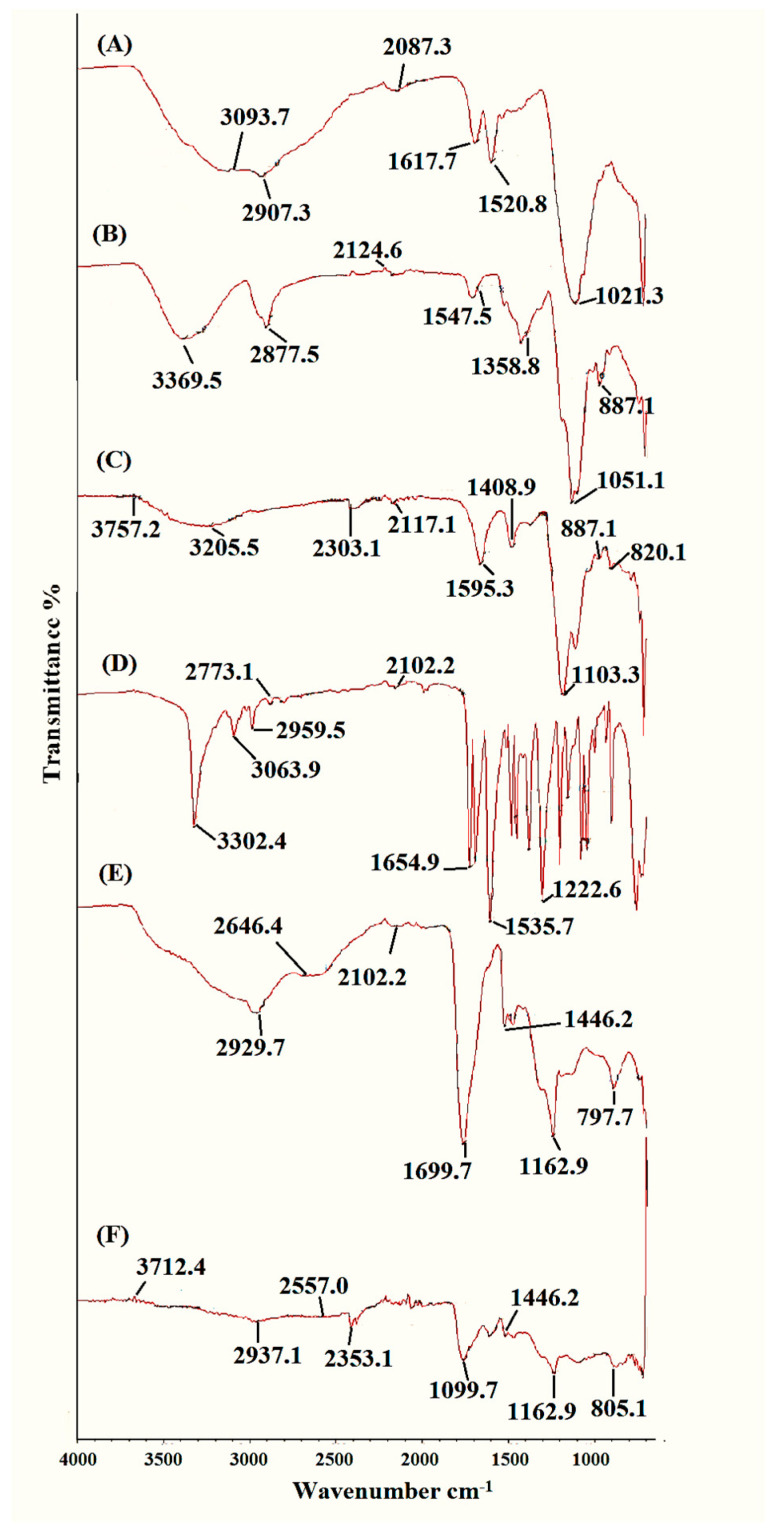
FTIR spectra of (**A**) neomycin; (**B**) HEC; (**C**) SA; (**D**) MBA; (**E**) HEC, SA, and MBA blend; (**F**) drug-loaded polymer blend.

The FTIR spectrum of polymers, drug, unloaded formulation, and drug-loaded formulation was analyzed for possible interaction and/or polymer–drug compatibility. The spectra are given in Figure 2A–F. The FTIR spectrum of neomycin sulfate (Figure 2A) showed a broad band at 3093.7 cm^−1^ which reflects the stretching frequencies of the NH_2_ group. The characteristic peak at 1617.7 cm^−1^ indicated the presence of the carbonyl functional group (–C=O), whereas the absorption band at 1520.8 cm^−1^ was assigned to aromatic C=C stretching [18].

HEC (Figure 2B) exhibited a broad band at 3388.5 cm^−1^, typically representing the stretching vibrations of the bound hydroxyl group (O-H) [19]. A sharp absorption peak at 2877.5 cm^−1^ was observed, which may be attributed to the vibrational stretching of the aliphatic C-H functional group. The absorption bands seen at 1647.5 cm^−1^ and 1358.8 cm^−1^ reflect the stretching vibrations of the glucose ring present in the structure of HEC. The C–O–C bonds are responsible for the stretching vibrations at 1051.1 cm^−1^, whereas the absorption band at 887.1 cm^−1^ represents the b-glucosidic linkages between the glucose rings [20].

The IR spectra of SA displayed vibrational stretching at 3205.5 cm^−1^, which corresponded to the stretching of the O-H functional group, as shown in Figure 2C. The characteristic peak at 1696.3 cm^−1^ was ascribed to the asymmetric stretching vibrations of the –COO− group present in the polymeric backbone of SA, whereas the peak at 1408.9 cm^−1^ represented the symmetric stretching vibrations of the –COO− group. A very sharp peak at wavenumber 1103.3 cm^−1^ indicated the presence of the C–OH group. The absorption band at 887.1 cm^−1^ was attributed to the vibrational stretching of the Na–O bond [21].

In the case of MBA, a sharp peak at 3302.4 cm^−1^ represented the stretching vibrations of the N–H functional group, as seen in Figure 2D. The absorption band at 1654.9 cm^−1^ was assigned to the vibrational stretching of the carbonyl group (–C=O). The strong absorption at 1535.7 cm^−1^ corresponded to the deformation of the N-H group. The existence of the C-N functional group in the structure of MBA was confirmed by a sharp peak at wavenumber 1304.6 cm^−1^. In addition, the absorption band at 670.9 cm^−1^ was seen due to the in-plane bending vibrations of the amide (O=C–N) group [22].

Distinctive peaks of HEC, SA, and MBA were found in the spectrum of the unloaded patch (Figure 2E), without any significant changes. The characteristic peak at 2383.1 cm^−1^ due to aliphatic C-H stretching was shifted to a higher intensity of 2929.7 cm^−1^. A very sharp peak at 1699.7 cm^−1^ was observed in the spectrum, attributable to the vibrational stretching of the carbonyl groups in polymers. The absorption band at 877 cm^−1^ due to the Na-O bond was relocated to a lower wavenumber of 797.7 cm^−1^. HEC and alginate bands were present in the FTIR spectra of the drug-free hydrogel formulation with minor changes. These changes signify the hydrogen bonding between the O-H group of HEC and the O-H of the COOH group of SA, thus leading to the formation of a polymeric cross-linked hydrogel system [23].

In comparison to the individual components, the FTIR spectrum of the drug-loaded hydrogel formulation showed a shift to a new distinct pattern, which might be attributed to the development of a new polymeric cross-linked hydrogel network. However, distinctive peaks of HEC, SA, MBA, and neomycin were also found, as observed in Figure 2F. A large valley formed in the range of 3600 to 3200 cm^−1^ when the –O–H stretching vibrations of polymers were overlapped by the -N-H stretching vibrations of the drug. The stretching at 3388.5 cm^−1^ due to the (-O-H) group was shifted to a higher frequency of 3520.1 cm^−1^_,_ indicating strong hydrogen bonding interactions between the (-O-H) group of HEC and the (-O-H) of the -COOH group of SA. Similarly, the (-C=O) stretching of the drug at 1617.7 cm^−1^ was overlapped by the vibrational stretching of the carbonyl group (-C=O) of polymers [23].

### 2.4. Differential Scanning Calorimetry (DSC) and Thermograviometric Analysis (TGA)

**Figure 3:**
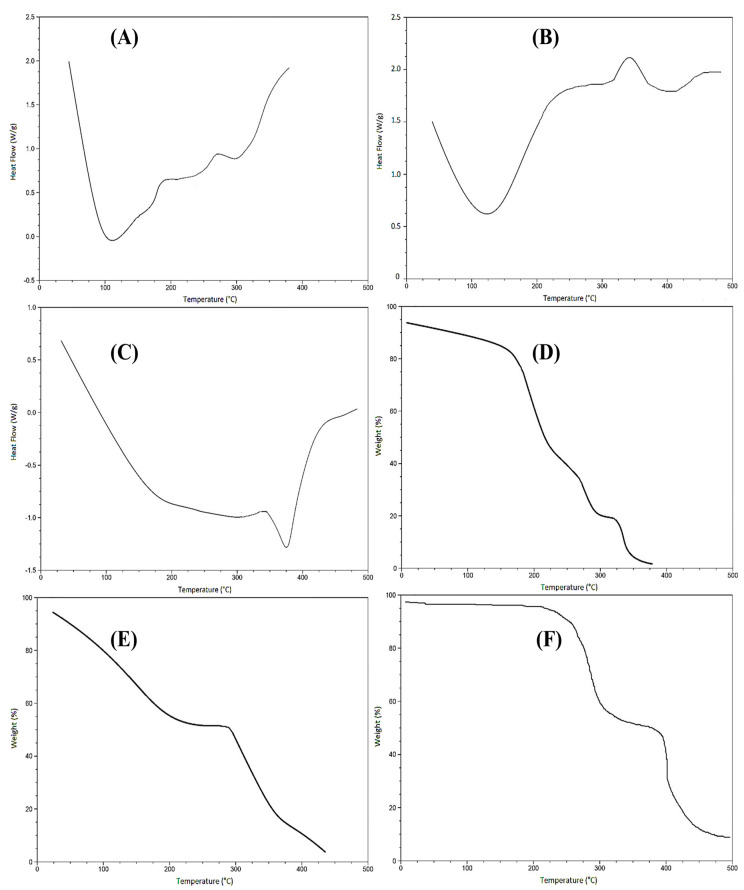
DSC graphs of (**A**) HEC, (**B**), SA, and (**C**) polymer blend. TGA curves of (**D**) HEC, (**E**) SA, and (**F**) polymer blend.

The DSC thermogram of HEC exhibited one endothermic and two exothermic peaks, as seen in Figure 3A. The endothermic peak, seen at 110 °C, is known as the glass transition temperature (Tg) corresponding to the loss of water molecules from the polymer. The first exothermic peak was observed at 189 °C, while the second exothermic peak was seen at around 270 °C, which might be due to the breakdown of polysaccharide chains indicating the melting point of HEC. Above 370 °C, all products of degradation were completely combusted [24]. The DSC thermogram of SA (Figure 3B) revealed an endothermic peak at 120 °C due to the loss of water molecules. An exothermic peak was also seen at 340 °C corresponding to the decomposition of polymeric chains [25]. The DSC thermogram of the polymeric cross-linked hydrogel formulation (Figure 3C) exhibited only one endothermic peak. This forward shift in endothermic peaks as compared to the individual polymer confirmed the formation of a more thermally stable formulation [24].

The thermograms of HEC, SA, and their blend are shown in Figure 3D–F, respectively. The decomposition of HEC started at 170 °C, contributing to 36% of weight loss in the region of 170–225 °C. This might be due to the breakdown of the polymeric backbone. The second step of thermal degradation took place in the area of 225–380 °C, corresponding to a weight loss of 43% due to the loss of water molecules from the hydrophilic groups of polymers. This step accounts for the devolatilization of the polymeric chains [26].

The TGA curve (Figure 3E) signifies that the degradation of SA took place in three steps. In the first phase, about 45% of the thermal degradation of SA takes place in the temperature range of 25–225 °C. This accounts for the breakdown of glucuronic and mannuronic acid residues in the polymeric chain. Decomposition below 200 °C describes the loss of moisture. In the second phase, thermal instability occurs in an area of 230–285 °C. The third stage of thermal decomposition occurs in the temperature range of 290–430 °C, and nearly 47% of the polymer was lost, indicating moisture loss and polymer chain breakdown. At 435 °C, the sample left a residual weight of about 4% [27]. The thermal degradation of the optimized formulation had two distinct phases of weight loss, as seen in Figure 3F. In the first phase, a weight loss of 40% occurred at 220 °C and ended at 300 °C due to the loss of moisture. The second stage of thermal degradation accounted for 45% of the weight loss and occurred in the region of 310 °C to 500 °C, corresponding to the breakdown of polymeric chains.

#### XRD Analysis of Topical Hydrogel Patch

**Figure 4:**
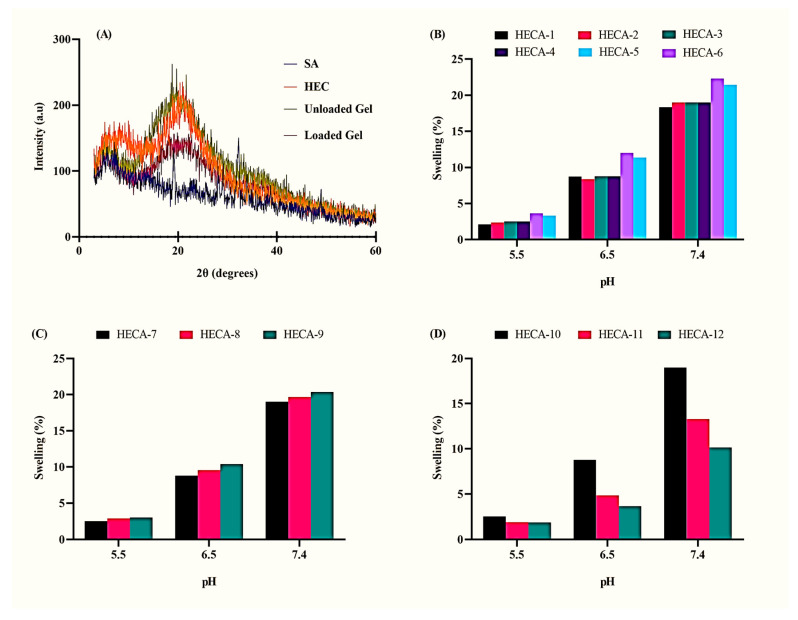
(**A**) XRD pattern of polymers and topical hydrogel patch. Dynamic swelling studies at different pH and concentrations of (**B**) polymer, (**C**) monomer, and (**D**) cross-linker.

The XRD analysis of HEC, SA, and formulation is shown in Figure 4A. The XRD pattern of SA showed a variety of sharp peaks equal to 19.15°, 29.15°, 32.25°, and 38.7°, which indicated the semi-crystalline nature of untreated SA [28]. The characteristic peak of HEC was observed at 20.4°, indicating the amorphous nature of untreated HEC [29]. The XRD pattern of the SA-HEC-based formulation showed only two sharp peaks at 19.7° and 21.65°, which confirmed the presence of SA and HEC in the developed formulation, respectively. The minor shifting of peaks was due to the formation of chemical bonds between HEC and SA polymers during polymerization. In general, HEC-SA-based polymeric cross-linked topical hydrogel formulations demonstrated an amorphous nature, which contributes to easy swelling and release of drug from the polymeric cross-linked hydrogel matrix.

### 2.5. Dynamic Swelling Studies of Topical Hydrogel Patch

Dynamic swelling studies were carried out at different pH (5.5, 6.5, and 7.4) to analyze the pH-sensitive behavior of the prepared hydrogel patches. The pH of the swelling medium is an important factor that influences the swelling properties of a hydrogel. In addition, swelling can also be affected by varying the concentrations of the polymer, monomer, and cross-linker [30]. As shown in Figure 4B, enhanced swelling behavior was observed when the amount of HEC was increased from HECA-1 to HECA-3. This behavior can be explained by the presence of a large number of hydrophilic groups in the structure of HEC, which also increases the drug-loading capacity of the hydrogel, thereby enhancing drug release [31]. In contrast, the mechanical integrity of patches with a higher concentration of SA (HECA-4 to HECA-6) weakened as the swelling behavior increased. The presence of several negatively charged carboxylate groups (-COO) on the Na-SA structure, which induces repulsion, can be used to explain this increase in swelling [32].

HECA-7, containing the highest content of AA as a monomer, exhibited the highest swelling as compared to HECA-5 and HECA-6, as given in Figure 4C. The higher concentration of AA increases the number of carboxylic acid groups (-COOH) for ionization, thus creating repulsion between polymeric networks and causing packed molecules to expand [33]. In a similar setting, greater swelling behavior was observed at pH 7.4 as compared to pH 5.5 and pH 6.5. At pH 5.5, molecules are tightly packed, but as the pH of the medium increases above 6, AA produces a greater number of negatively charged carboxylate ions (-COO). This creates repulsion between chains of the polymeric matrix, thereby expanding them, and as a result, the dynamic swelling increases [34]. HECA-12, containing the highest concentration of MBA as a cross-linker, exhibited the least swelling dynamics as compared to HECA-10 and HECA-11 (Figure 4D). It has also been reported in the literature that an increase in cross-linking agents increases the intermolecular forces between polymers. This leads to the formation of a more rigid and strong polymeric matrix, which results in less expansion of chains, thus reducing the penetration of water molecules. As a result, hydrogel loses its elasticity and results in a low percentage of swelling [35].

### 2.6. Sol–Gel Analysis

**Figure 5:**
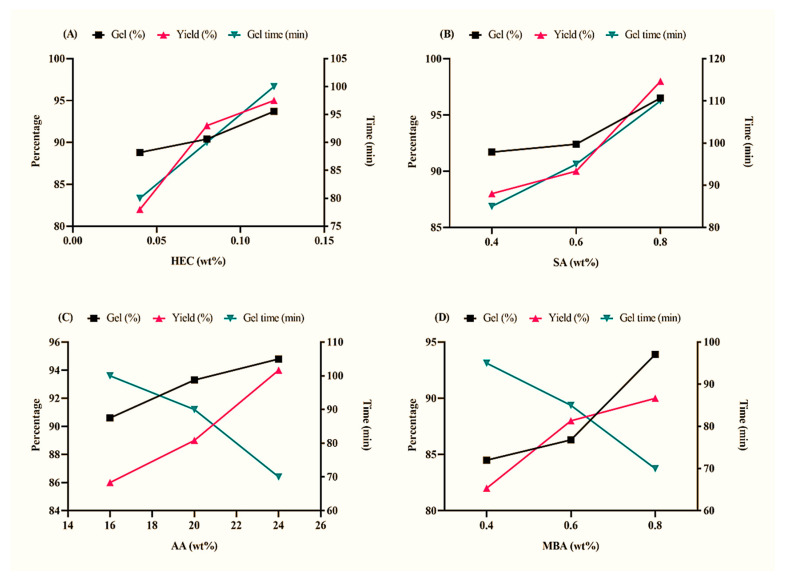
Gel (%), yield (%), and gelling time (min) of (**A**) HEC, (**B**) SA, (**C**) AA, and (**D**) MBA.

Sol–gel analysis was carried out to determine the fractions of unreactive components, including polymers, monomers, and cross-linkers, left during the development of a hydrogel topical patch. It was observed that the sol (%) decreased with an increase in the concentration of such components. Alternatively, it can be safely said that the gel fraction increased along the formulation series when the concentration of HEC, SA, AA, and MBA increased. This proves that an efficient formation of a cross-linking network occurred under the adopted manufacturing conditions [36]. The percentages of gel and yield shown in Figure 5A,B were proportional to the amount of polymer. This can be attributed to the production of macromolecule radicals during the polymerization reaction [37]. Such a case was true for both HEC (HECA-1 to EHCA-3) and SA (HECA-4 to HECA-6) formulations. However, with an increase in polymer concentration, more time was required to form the gel due to the limited availability of MBA to cross-link the monomers in the hydrogel network. As can be seen in Figure 5C, an increase in AA concentration led to enhanced gel (%) and yield (%) due to the formation of a large number of free radicals. Alternatively, decreased gel time was observed, which can be attributed to a faster reaction rate at higher monomer concentrations [38]. Similar results were obtained with MBA concentration (Figure 5D), whereby the increase in the concentration of the cross-linker increased the gel (%) and yield (%) and decreased the gel time [37].

### 2.7. In Vitro Drug Release Evaluation 

**Figure 6:**
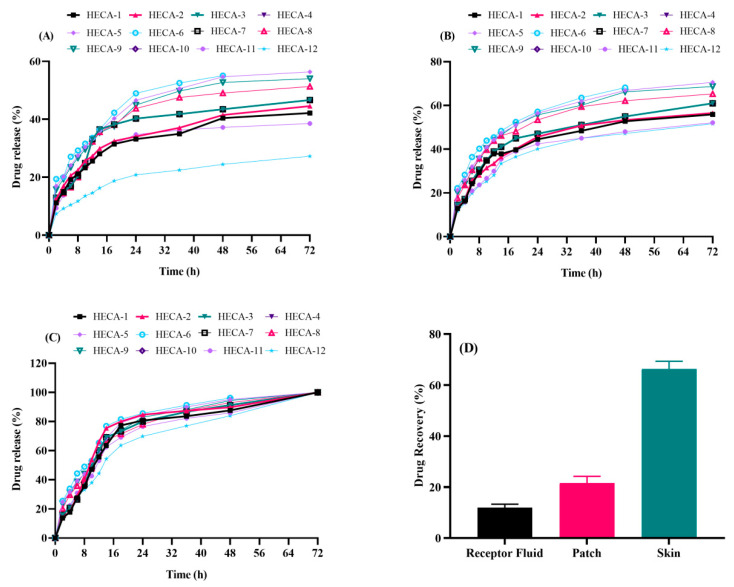
Drug release (%) of neomycin from topical patch at (**A**) pH 5.5, (**B**) pH 6.5, and (**C**) pH 7.4. (**D**) Drug deposition in skin after ex vivo analysis.

The in vitro release tests were conducted at pH 5.5 for normal skin and pH 6.5 and 7.4 for injured skin [39]. Figure 6A shows that with an increase in HEC concentration, the drug release pattern increased from HECA-1 to HECA-3. The existence of a large number of hydrophilic groups in the polymer’s structure, which interact with the surrounding solvent, can be used to explain this increase in drug release. At constant monomer and cross-linker contents, SA (HECA-4 to HECA-6) showed a similar rise in drug release. Because of the presence of a greater number of available carboxylic groups on the structure of SA, HECA-6 demonstrated the highest drug release.

Similarly, an increased monomer concentration resulted in a change in the drug release pattern in formulations from HECA-7 to HECA-9 (Figure 6B). The explanation for enhanced drug release is that AA increases the number of carboxylic acid groups available for ionization, producing repulsion between the polymeric networks and forcing packed molecules to expand. Because there were many negatively charged carboxylate ions present at pH 7.4, greater drug release was seen compared to pH of 5.5 and 6.5.

On the other hand, as the cross-linker concentration was increased, the drug release pattern decreased in formulations from HECA-9 to HECA-12 as shown in Figure 6C. As the MBA concentration rises, so do the intermolecular forces between polymers, creating a more rigid and robust polymeric matrix and reducing chain expansion. As a result, the hydrogel becomes less elastic and releases a lesser amount of drug [36].

### 2.8. Kinetic Modeling for Drug Release Profile

The release profile of neomycin was analyzed by different kinetic models to understand the pattern of drug release from the topical hydrogel patch. As seen in Table 2, the formulation followed zero-order kinetics, signifying that the drug release was independent of the initial drug concentration [40]. The release of drug from the hydrogel system is a complicated process; hence, the KP model was used to identify the drug release mechanism. As affirmed in Table 2, the *n* value for most of the formulations was less than 0.5, which indicated a diffusion-based drug release from the topical patches [40]. However, HECA-1, HECA-3, HECA-4, HECA-10, and HECA-12 favored anomalous drug release, demonstrating both diffusion and erosion mechanisms [17].

The drug release from the hydrogel matrix can be divided into three major phases. To begin with, drug-loaded hydrogel formulations have relatively little water and porosity, restricting drug mobility. In the second step, water molecules penetrate the hydrogel network, causing the polymeric chains to relax, increasing porosity and increasing drug release. Finally, after being hydrated, the polymeric network has maximal porosity and releases the medication to its full potential [41]. 

### 2.9. Ex Vivo Skin Drug Deposition Studies

As per ex vivo analysis on the Franz diffusion cell, only 12,948 μg (12%) of drug permeated into the receptor compartment over an area of 1.5 cm^2^ after 72 h. Afterwards, the skin was demounted and analyzed for drug deposited in the skin. As seen in Figure 6D, 23,332 μg remained on the skin surface (21.62% of the total drug). These data clearly suggested that neomycin tended to retain in the skin layers, and a little amount was available transdermally. This created increased residence of the drug on the skin surface, which is ideal for topical drug delivery in order to treat wounds and skin infections.

### 2.10. Primary Skin Irritation Test

**Figure 7:**
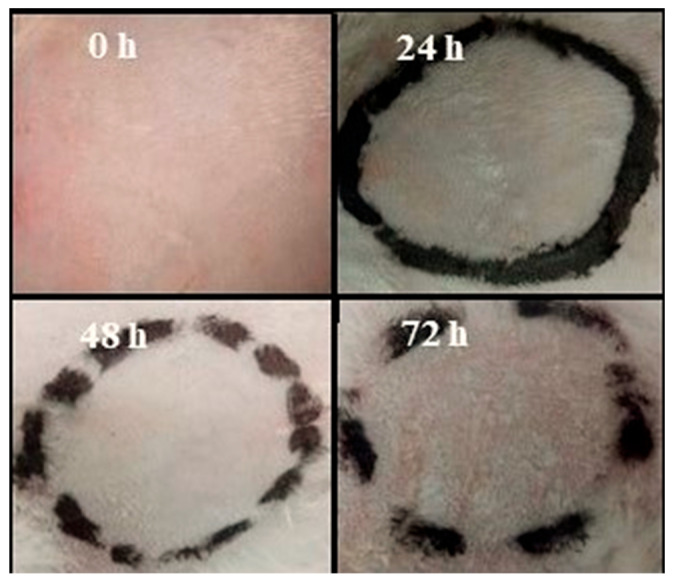
Skin irritation analysis using Draize test.

The Draize scale revealed no signs of irritation, inflammation, or erythema on rabbit skin, as evident in Figure 7, implying that all components of polymeric cross-linked topical hydrogel patches are safe. As a result, the created hydrogel patches demonstrated greater skin tolerability for improved wound healing and skin infection prevention, thus covering all features of an ideal wound dressing material.

### 2.11. In Vivo Wound-Healing Potential

**Figure 8:**
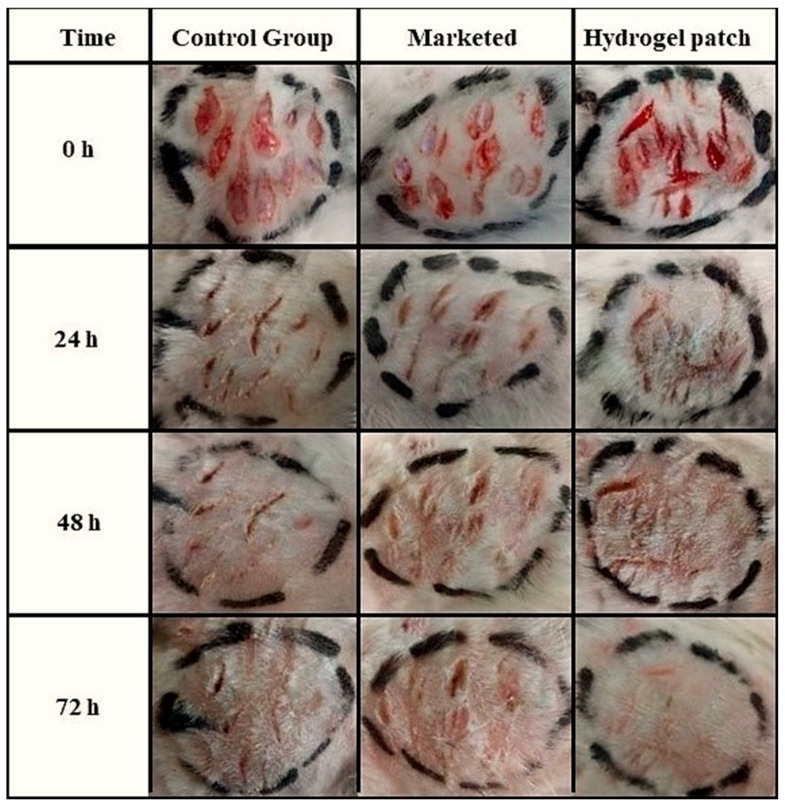
In vivo wound healing using neomycin hydrogel patch and marketed cream formulation.

To check the degree of wound healing, the rabbits were observed every day for three days. The results showed that neomycin hydrogel patches had fast healing potential as compared to the marketed formulation, as confirmed by the photograph in Figure 8. In contrast to conventional topical wound treatments, the formulated hydrogel patches retained a large amount of water content because of their hydrophilic polymers. HEC and SA kept the wound bed moist and absorbed all exudate to prevent the formation of edema at the wound site. In addition, neomycin prevents the entry of pathogens at the site of the wound along with a natural polymer (SA), which itself has tissue-repairing ability and also accelerates the healing process.

## 3. Conclusions

The free radical polymerization approach was used to successfully create polymeric cross-linked topical hydrogel patches based on natural SA and synthetic HEC polymers. The development of a new and stable topical hydrogel patch with good mechanical strength has been confirmed by all characterization investigations. The formulation’s stability was further demonstrated by FTIR, DSC, TGA, and XRD. The formulations with the highest swelling dynamics and drug release were those with the highest concentrations of polymers and monomers and the lowest concentration of cross-linker. All formulations demonstrated pH-sensitive behavior. The kinetic study of hydrogel patches revealed that all formulations used zero-order kinetics. A wound healing study found that neomycin-loaded hydrogel patches heal faster than commercial formulations due to the inclusion of natural wound-healing polymer (SA), which promotes epithelialization and collagen formation. The formulated hydrogel patches will be used in the near future to heal wounds, especially dry wounds, due to the creation of a damp environment that removes dead tissues and foreign materials from the wound. The patches will provide a biological environment to the wounds and will prevent them from any antimicrobial agents because of having their own antimicrobial properties.

## 4. Materials and Methods

### 4.1. Materials 

Sodium alginate (SA), hydroxyethylcellulose (HEC), N,N′-methylenebisacrylamide (MBA), ethanol, and ammonium persulfate (APS) were purchased from Merck, Germany. Acrylic acid (AA), sodium hydroxide (NaOH), potassium dihydrogen phosphate, and hydrochloric acid were purchased from Sigma Aldrich, UK. All reagents were of analytical grade. Neomycin sulfate was humbly gifted by Atco Laboratories, Karachi, Pakistan.

### 4.2. Methods

#### 4.2.1. Preparation Method of Topical Hydrogel Patch

The methodology utilized for the preparation of polymeric cross-linked topical hydrogel patches was the free radical polymerization technique [12]. The chemicals, at the specified quantities given in Table 3, used in the synthesis of topical hydrogel patches include SA and HEC as polymers, AA as a monomer, APS as an initiator, and MBA as a cross-linker. The required quantity of SA was dissolved in 5 mL of distilled water and stirred for 20 min on a hot-plate magnetic stirrer at a temperature of 37 °C to get a homogenous solution. Similarly, HEC was individually dissolved in 5 mL of distilled water, and a clear solution was obtained by stirring it on a hot plate at a temperature of 90 °C. A specified amount of APS and MBA was taken in a separate beaker and dissolved in 5 mL of distilled water on a hot-plate magnetic stirrer at 37 °C to get a clear solution. AA was taken separately in a beaker. The prepared solutions were poured into the cooled polymer mixture drop-wise. The prepared reaction mixture was carefully poured into labeled Petri dishes, covered with aluminum foil, and placed in a preheated electronic water bath at 50 °C for 2 h, then at 60 °C for 2 h, and then at 65 °C overnight. After a period of 24 h, patches were removed from the Petri dishes and washed gently with a water–ethanol mixture (70:30) to remove uncross-linked chemicals and impurities [12]. Washed hydrogel patches were dried in a vacuum oven at 40 °C for 2 days. After drying, polymeric cross-linked hydrogel patches were stored in polythene bags.

For the macro-appearance of the hydrogel patch, the formulation was observed after drying of the formulation. For the microscopic analysis using SEM, the fabricated hydrogel patches were dried at 40 °C in an electric oven before being crushed and ground into small pieces with a pestle and mortar.

#### 4.2.2. Macro-Morphological and Micro-Morphological Evaluation of Topical Hydrogel Patch

For the macro-appearance of the hydrogel patch, the formulation was observed after drying of the formulation. For the microscopic analysis using SEM, the fabricated hydrogel patches were dried at 40 °C in an electric oven. The patches were made swollen to enhance the appearance of the porous structure. The samples were then completely dried, crushed, and ground into small pieces with a pestle and mortar. Double-sided tape was used to adhere a gold-coated aluminum stub, and the tape was covered with a weighed sample of finely divided powder. The aluminum stub was then positioned in the vacuum chamber of the microscope and morphologically assessed using an electron beam. A photomicrograph examined under a microscope was used for the morphological characterization of the topical patch [39].

#### 4.2.3. Physical Characteristics of Topical Hydrogel Patches

The hydrogel patches were evaluated for physical characteristics including thickness, weight, and folding endurance. For the thickness of the patch, a digital vernier caliper was used, and an average of three formulations was taken. The weight of each patch in triplicate was taken on a digital weighing balance. In the case of folding endurance, the patch was folded from the same position 400 times. The count at which the patch broke or lost its strength was noted as the folding endurance (n = 3) [40].

#### 4.2.4. Characterization of Topical Hydrogel Patch for Polymer Compatibility and Thermal Stability

FTIR technique was used to describe the presence of various functional groups in molecular structure. It is used to determine the cross-linking mechanism for pure material and specific impurities in developed smart gels. The structural arrangement of polymeric smart gels was evaluated and analyzed by attenuated total reflectance–Fourier transform infrared spectroscopy (Tensor 27 series, Germany) in the 4000–650 cm^−1^ range [15].

Thermal analysis was conducted using differential scanning calorimetry (DSC) and thermogravimetric analysis (TGA) (TA instruments Q5000 series, West Sussex, UK). For TGA analysis, a 0.5–5 mg sample was placed in an open platinum 100 µL pan that was attached to a microbalance and heated to 20–600 °C under dry nitrogen. For DSC, the sample was placed into an aluminum pan and heated with nitrogen gas at a rate of 20 °C/min [39].

XRD study was performed at room temperature by using a Bruker D-8 powder diffractometer (Bruker Kahlsruhl, Germany). A copper-Kα radiation source was adjusted at a wavelength of 1.542 Å and 1 mm slits, and the pure polymer and the prepared gel sample were investigated (10–80° 2θ) at a rate of 1 °C/min [42].

#### 4.2.5. Sol–Gel Fraction

The polymeric cross-linked hydrogel patches were dried in an electric oven at 40 °C. After drying, the patches were weighed on an electronic balance and transferred individually into labeled beakers containing 200 mL of distilled water. Occasional stirring was required to remove uncross-linked polymer components from the patch structure. After 24 h, hydrogel patches were removed from distilled water, transferred into labeled Petri dishes, and placed in the oven for drying to get a constant weight. The gel and sol fraction was calculated by Equations (1) and (2), respectively.

Gel (%) = [Wg/W0] × 100(1)

Sol (%) = 100 − Gel (%)(2)

where the initial weight of the dried patch is W0, and the weight of the extracted dried patch is Wg [43]. 

#### 4.2.6. Dynamic Swelling Studies of Topical Patch

In order to measure the pH-sensitive behavior of hydrogel patches, a dynamic swelling study was carried out at different pH levels (5.5, 6.5, and 7.4) using phosphate buffer. The buffer was placed in a 250 mL beaker and labeled individually for each formulation (n = 3). The pre-weighed patches were placed in buffer solution for 72 h. After regular time intervals, the patches were taken out of respective buffer solutions and swabbed with the help of blotting paper to remove excessive buffer. The accurately weighed patches were then transferred to buffer solution, and the whole process was repeated unless a constant weight was attained. The degree of swelling was calculated in grams by Equation (3):

Swelling at time t = (mt − m0)/m0(3)

where mt is the weight after time t, and m0 is the initial weight at time zero.

#### 4.2.7. Neomycin-Loaded Topical Hydrogel Patch

Drug loading in a polymeric cross-linked hydrogel patch was carried out by the swelling diffusion technique. For this, neomycin sulfate (1 g) was weighed on a digital balance and transferred into a beaker containing phosphate buffer (pH 7.4, 200 mL). A homogenous drug solution was prepared by continuous stirring on a hot-plate magnetic stirrer. After accurately weighing the formulated patches, they were immersed in the drug solution for a period of 24 h at a temperature of 37 °C. Neomycin hydrogel patches were withdrawn from the drug solution after the required period of time, and impurities were removed by washing with distilled water. Drug-loaded patches were dried in an electric oven at 40 °C and weighed again. The drug-loaded hydrogel patch will remain intact on the wounded skin and will release the loaded drug for a prolonged time until the maximum amount of drug is made available for its therapeutic effect. The drug-loaded patch can be removed immediately if any adverse effects are observed.

#### 4.2.8. In Vitro Drug Release Evaluation

Neomycin-loaded hydrogel patches were weighed on a digital weighing balance and transferred in baskets of dissolution apparatus containing 900 mL of phosphate buffer of different pH (5.5, 6.5, and 7.4) at a temperature of 37 °C ± 0.5 °C. The release assay was conducted under sink conditions. The paddle was rotated at 50 rpm, and a 5 mL aliquot was taken from the dissolution medium after a specific time interval. In order to maintain the sink condition, 5 mL of fresh medium was added to the main dissolution basket. The samples were filtered and appropriately diluted, and absorbance was measured on UV–visible spectrophotometer (UV-1601 Shimadzu, Japan) at 208 nm [44]. The amount of drug released in the buffer was calculated with reference to the calibration curve in the phosphate buffer (y = 0.0138x − 0.0355; R² = 0.9987 in pH 5.5, y = 0.0109x − 0.0276; R² = 0.9981 in pH 6.5, and y = 0.0147x − 0.0235; R² = 0.9991 in pH 7.4). The samples were processed in replicates three times through the UV spectrophotometer, and the average absorbance of the samples was noted.

#### 4.2.9. Release Kinetic Study of Ivabradine HCl

In vitro dissolution studies were further examined using a model-dependent methodology by fitting the results to the following models:

(4) $$\mathrm{Zero}-\mathrm{order}\;\mathrm{kinetics}=Q_{t}=Q_{o}+K_{0}$$

(5) $$\mathrm{First}-\mathrm{order}\;\mathrm{kinetics}\;=\log Q_{t}=\log Q_{o}+K_{1}t/\;2.303$$

(6) $$\mathrm{Higuchi}\;\mathrm{equation}=M_{t}/M_{\infty \;}=k_{t}\surd t$$

(7) $$\mathrm{KorsmeyerPeppas}\;\mathrm{equation}\;=M_{t}/M_{\infty \;}=k_{3}t^{n}$$

where *Q_t_*, amount of drug dissolved in time *t*; *Q_o_*, initial amount of drug in the solution; *K*_0_, zero-order release constant; *K*_1_, first-order release constant; *M_t_*, cumulative amount of drug released at time *t*; *M*, absolute cumulative amount of drug released at infinite time; *k*_2_, constant reflecting the design variable of the system; *k*_3_, constant incorporating structural and geometric characteristics of the device; *n*, release exponent indicative of the mechanism of drug release [40,45].

#### 4.2.10. Ex Vivo Skin Deposition Analysis

Ex vivo skin deposition studies were carried out according to the protocol described by Shabbir et al. (2016) with slight modifications [40]. A Franz diffusion cell was used to evaluate the ex vivo drug deposition of neomycin in excised rabbit skin. The dermal side of the skin was positioned onto the receptor compartment. A circular hydrogel patch was placed on the skin and secured with the clamps. The receptor compartment was filled with phosphate buffer. The apparatus was linked to a thermostatically controlled water bath, which circulated water via a jacket enclosing the cell body to keep the temperature at 32 ± 2 °C. After 72 h, the patch was removed from the skin surface, and the amount of drug retained on the surface was estimated after washing. The drug deposited in the skin was calculated by subtracting the surface drug and receptor compartment drug from the initial drug concentration of neomycin in the patch [15].

#### 4.2.11. Primary Skin Irritation Test

Before applying the neomycin hydrogel patch to the rabbit skin, a basic skin irritation test was performed to guarantee that no harm would be done to the skin under normal conditions. The hairs from the rabbit skin were removed according to the protocol described by Nagra et al. (2022) [15]. For this, white albino rabbits (1.5 to 2 kg body weight) were bought from an animal house at the Faculty of Pharmacy, University of Lahore. The study was conducted according to ICH guidelines and as per protocols designed by Institutional Research Ethics Committee (IREC). Permission for the conduct of the study was granted by IREC under reference number IREC-22-17. Rabbits (n = 9) were equally divided into three groups, namely Group I (Control group), Group II (marketed neomycin topical cream), and Group (III) (Neomycin topical patch). The patch was placed on an accessible area, most notably the abdomen, and wrapped with tape. The experiment was carried out over the course of three days. The Draize patch test scale was used to assess skin response. Rabbits were examined for signs of irritation, inflammation, and erythema. The degree of the reaction was measured by assigning a score of 0 (no reaction), 1 (minor reaction), 2 (well-defined reaction), 3 (moderate reaction), and 4 (severe scar development) [46].

#### 4.2.12. In Vivo Wound-Healing Analysis

A wound-healing study was carried out to observe the healing capability of neomycin hydrogel patches. For this, albino rabbits (n = 9) were equally divided into three groups, namely Group I (Control group), Group II (marketed neomycin topical cream), and Group (III) (Neomycin topical patch). Before the start of experimentation, the rabbits were given local anesthesia, and minor superficial cuts were applied to the skin with the help of a sterilized blade, and the area was marked. No treatment was applied to control Group I, and the wound was kept open. Group II and Group III received the medicament. The study was continued for 3 days, and rabbits were observed after 24, 48, and 72 h of application.

## Figures and Tables

**Table 1:** Physicochemical properties of polymeric cross-linked topical hydrogel patches.

Formulation	Thickness (mm)	Weight Variation (g)	Folding Endurance
**HECA-1**	1.75 ± 0.05	2.41 ± 0.30	333 ± 20
**HECA-2**	1.78 ± 0.13	2.43 ± 0.21	342 ± 15
**HECA-3**	1.80 ± 0.07	2.47 ± 0.16	348 ± 08
**HECA-4**	1.82 ± 0.18	2.49 ± 0.24	350 ± 12
**HECA-5**	1.86 ± 0.11	2.65 ± 0.09	356 ± 16
**HECA-6**	1.89 ± 0.09	2.76 ± 0.15	362 ± 23
**HECA-7**	1.83 ± 0.16	2.50 ± 0.10	355 ± 21
**HECA-8**	1.91 ± 0.05	2.81 ± 0.07	368 ± 12
**HECA-9**	1.95 ± 0.08	2.94 ± 0.16	374 ± 09
**HECA-10**	1.81 ± 0.11	2.48 ± 0.13	335 ± 16
**HECA-11**	1.85 ± 0.06	2.57 ± 0.18	315 ± 18
**HECA-12**	1.87 ± 0.02	2.62 ± 0.12	320 ± 10

**Table 2:** Kinetic mModeling on neomycin-loaded polymeric cross-linked hydrogel patch.

Formulations	Zero-Order	First-Order	Higuchi Model	Korsmeyer–Peppas	
	R^2^	R^2^	R^2^	R^2^	n
**HECA-1**	0.9865	0.6104	0.9627	0.9246	0.521
**HECA-2**	0.9801	0.5299	0.9475	0.8968	0.496
**HECA-3**	0.9854	0.5869	0.9644	0.9296	0.509
**HECA-4**	0.9858	0.5872	0.9646	0.9299	0.505
**HECA-5**	0.9896	0.2936	0.9804	0.9626	0.415
**HECA-6**	0.9893	0.1618	0.9735	0.9553	0.455
**HECA-7**	0.9855	0.5862	0.9645	0.9293	0.499
**HECA-8**	0.9952	0.4239	0.9847	0.9719	0.444
**HECA-9**	0.9934	0.3191	0.9848	0.9712	0.419
**HECA-10**	0.9862	0.5867	0.9644	0.9295	0.504
**HECA-11**	0.9929	0.5573	0.9762	0.9532	0.489
**HECA-12**	0.9949	0.6062	0.9856	0.9714	0.501

**Table 3:** Composition of hydrogel topical patch.

Formulation	SA (g)	HEC (g)	AA (g)	APS (g)	MBA (g)
**HECA-1**	0.1	0.01	4	0.1	0.1
**HECA-2**	0.1	0.02	4	0.1	0.1
**HECA-3**	0.1	0.03	4	0.1	0.1
**HECA-4**	0.1	0.03	4	0.1	0.1
**HECA-5**	0.15	0.03	4	0.1	0.1
**HECA-6**	0.2	0.03	4	0.1	0.1
**HECA-7**	0.1	0.03	4	0.1	0.1
**HECA-8**	0.1	0.03	5	0.1	0.1
**HECA-9**	0.1	0.03	6	0.1	0.1
**HECA-10**	0.1	0.03	4	0.1	0.1
**HECA-11**	0.1	0.03	4	0.1	0.15
**HECA-12**	0.1	0.03	4	0.1	0.2

## Data Availability

Not applicable.

## Abbreviations

SA: sodium alginate, HEC: hydroxy ethyl cellulose, AA: acrylic acid, MBA: N,N-methylene bisacrylamide, SEM: scanning electron microscopy, FTIR: Fourier transform infrared spectroscopy, XRD: X-ray diffraction, TGA: thermogravimetric analysis, DSC: differential scanning calorimetry.
